# A comprehensive review program to prepare pharmacy students for the Saudi Pharmacist Licensure Examination (SPLE)

**DOI:** 10.1016/j.jsps.2022.07.017

**Published:** 2022-07-30

**Authors:** Ibrahim Sales, Abdulrahman M. Alwhaibi, Raniah I. Aljadeed, Rawan F. Alzaidi, Ahmad Shahba, Mansour Almuqbil, Aws Alshamsan

**Affiliations:** a Department of Clinical Pharmacy, College of Pharmacy, King Saud University, P.O. Box 2454, Riyadh 11451, Saudi Arabia; b Department of Pharmaceutics, College of Pharmacy, King Saud University, P.O. Box 2454, Riyadh 11451, Saudi Arabia

**Keywords:** Saudi Pharmacists Licensure Examination, Licensing exam, SPLE preparation, Pharmacy education, Pharmacy licensure, Saudi Arabia

## Abstract

**Purpose:**

The Saudi Pharmacist Licensure Examination (SPLE) has been mandated by the Saudi Commission for Health Specialties (SCFHS) for three and a half years; however, colleges of pharmacy and/or pharmacy organizations in Saudi Arabia have yet to implement a comprehensive review program to help prospective graduates to succeed. The aim of this study was to assess the impact of an integrated program designed to enhance students’ performance on the SPLE.

**Methods:**

A cross-sectional study was conducted to determine the impact of integrating SPLE review activities (clinical review quizzes (CRQs), disease state presentations (DSPs), a Capstone OSCE, and a mock SPLE) on students’ SPLE results and perceptions of their SPLE preparation and performance. Student scores from the review activities were analyzed and an anonymous, voluntary survey was used to assess the impact of these activities on student readiness levels and performance on the SPLE.

**Results:**

A total of 127 Doctor of Pharmacy (Pharm.D.) students were included in the study. The average scores for the mock SPLE, DSPs, and Capstone OSCE were (55.8% ± 8.55), (91.3% ± 7.17), and (95.2% ± 6.90), respectively. Approximately 50% of the students responded to the survey. Most students had taken and passed the SPLE on the first attempt (94.6%) with an average score of 635.7 ± 39.4 (∼79%). Over 60% and 70% of students recommended the SPLE CRQs activity and the DSP activity, respectively. With respect to mock SPLE, 60.9% believed that it provided an idea of what to expect on the SPLE and 54.7% recommended to add the Capstone OSCE into the curriculum. Overall, the majority of students would recommend these activities be incorporated in the college of pharmacy curriculum in order to better prepare pharmacy graduates for the SPLE.

**Conclusion:**

Prospective graduates from Saudi universities may benefit from college of pharmacy-organized SPLE reviews. Based on this study’s findings, a comprehensive review course including a mock SPLE and disease state presentations is recommended. In addition, SPLE review lectures, CRQs, and a Capstone OSCE may provide additional benefit.

## 1 Introduction

In January 2019, the Saudi Commission for Health Specialties (SCFHS) mandated the Saudi Pharmacist Licensure Examination (SPLE) for entry-to-practice pharmacy graduates from local universities. Before that date, passing the pharmacy licensure exam was obligatory only for those who graduated from non-Saudi universities and wanted to practice in Saudi Arabia. In order to draft the initial exam blueprint, a panel of experts was assembled representing multiple areas of pharmacy expertise. The panel followed a systematic approach including benchmarking, surveys, brainstorming, focus groups, and expert judgment. In conclusion, the panel developed a SPLE blueprint consisting of four pharmacy content areas: Basic Biomedical Sciences (10%), Pharmaceutical Sciences (35%), Social/Behavioral/Administrative Sciences (20%), and Clinical Sciences (35%).(Saudi Commission for Health Specialties, 2019) The exam consists of 300 multiple choice questions. Candidates are allotted 6 hours to finish the exam and must score ≥ 536 (∼67%) on a scale of 200–800 points in order to pass the exam. Each year, SCFHS releases the candidates’ performance on the SPLE from all colleges including passing rates on the first attempt, highest grades, and the mean score.

Besides the cost of retaking the exam, remediation after the first attempt may delay the candidates’ employment opportunities, preclude applying to residency program in the same year of graduation, as well as negatively impacting the college’s overall score.(Lebovitz et al., 2017) Therefore, many colleges have designed preparatory courses and workshops for their students. They also started to update their curricula to improve the first attempt passing rates of their students. These updates included changes in didactic courses and internship rotations, changing the assessment tools such as Objective Structured Clinical Examinations (OSCEs), and in some cases, a complete reform of the entire curriculum. (Hirsch and Parihar, 2014).

In addition to didactic lectures, diversified methods such as laboratory, critical appraisals, and simulation have been considered for inclusion in many pharmacy curricula. (Accreditation Council for Pharmacy Education, 2016, Elder et al., 2019) Some of these teaching methods became essential to obtain local and international academic accreditation. (Accreditation Council for Pharmacy Education, 2016, National Center for Academic Accreditation and Evaluation, 2018) Teaching method diversification has been found to be very helpful in preparing students for both Introductory Pharmacy Practice Experiences (IPPEs) and Advanced Pharmacy Practice Experiences (APPEs). Moreover, it will help pharmacy students to achieve the Program Learning Outcomes (PLOs) and prepare them for real-world practice. (Hirsch and Parihar, 2014, Phillips et al., 2019).

Several variables have been associated with candidate success on pharmacy licensure exams. These include gender, age, type of college (public or private), GPA score, preparatory year score, taking a mock exam, and participation in an integrated longitudinal activity.(Chisholm-Burns et al., 2017, Chisholm-Burns et al., 2014, Williams et al., 2019) Karimi et al. reported that students who participated in a longitudinal pharmacotherapy activity scored higher on their NAPLEX exams than their colleauges who did not participate.(Karimi et al., 2014) The study also found that students believed that having different curricula activities assisted them for preparation in licensure examination. Another study found that lab-based activities such as patient case presentations and associated critical thinking questions were associated with better performance on the licensure examination. (Elder et al., 2019).

In Saudi Arabia, one study has been published examining the associations between student factors and higher SPLE scores. A study conducted by Alhifany et al. revealed that the male gender, pharmacy GPA, and higher scores in pharmacology and therapeutic courses were associated with higher scores on the SPLE.(Alhifany et al., 2020) However, there has been no single study in Saudi Arabia that has assessed the impact of an integrated program designed to enhance students’ performance on the SPLE. The objectives of this cross-sectional study were to determine the impact of integrating SPLE review activities (clinical review questions (CRQs), disease state presentations (DSPs), a Capstone OSCE, and a mock SPLE) on students’ SPLE results and perceptions of their SPLE preparation and performance.

## 2 Methods

### 2.1 Study design

A cross-sectional study was conducted at the King Saud University (KSU) College of Pharmacy in Riyadh, Saudi Arabia, from January to May of 2020. It involved 127 Level 10-Doctor of Pharmacy (PharmD) students enrolled in the Pharmacy Practice Lab 6 (PPL-6) course. The PPL-6 course is the final lab that students take prior to their advanced pharmacy practice experience (APPE) internship year. The lab is taken simultaneously with the Oncology and Acute Care Pharmacotherapy modules and primarily focuses upon pharmacotherapy cases and activities related to those modules. In addition, the PPL-6 course historically incorporated activities and topics that were inadequately or not covered anywhere else in the PharmD curriculum. Various activities were integrated into the course syllabus for the SPLE review and included a mock SPLE exam, CRQs, group DSPs, and a Capstone OSCE. A one-hour overview presentation about the SPLE was presented to the students at the beginning of the semester.

### 2.2 Procedure

#### 2.2.1 Mock SPLE

A written mock SPLE exam was designed by KSU College of Pharmacy faculty members from different specialties. A bank of 200 mock SPLE questions was compiled. The exam questions were previously piloted on a sample of 130 students prior to the PPL-6 lab. Prior to the COVID-19 pandemic, the instructors planned to give a pre- and post-mock SPLE exam. The pre-mock SPLE consisted of 100 questions and covered the Basic Biomedical Sciences, Pharmaceutical Sciences, Social/Behavioral/Administrative Sciences, and Clinical Sciences domains. An entire lab session was dedicated to taking the pre-mock SPLE at the beginning of the semester before the students participated in any of the other SPLE preparatory activities. Exam monitors were assigned, and students were allowed 2 hours to complete the exam. The post-mock SPLE exam was canceled after a nationwide lockdown was implemented in Saudi Arabia due to the COVID-19 pandemic.

#### 2.2.2 SPLE CRQs

Students were required to individually answer and submit five NAPLEX review questions from a designated list of a disease states on a weekly basis for 10 weeks. The list included the most common disease states covered in the curriculum and overlapped with the NAPLEX Review Guide sections and the DSP schedule (Appendix A). Students were provided access to the McGraw-Hill Education 3rd Edition NAPLEX Review Guide through the Saudi Digital Library’s AccessPharmacy subscription. Permission to use the program was sought and granted by McGraw-Hill Education. The NAPLEX Review Guide features a quiz option which allows users to choose the number of questions desired and then the program randomly selects questions from a fixed bank of questions. Students in the PPL-6 course were assigned topics on a weekly basis and were required to answer five questions. Students could take the exam as many times as needed in order to obtain a score of at least 60%. The student must email his/her results to the responsible facilitator.

#### 2.2.3 Disease state presentations

Students were divided into groups of five or six students for the DSPs. An activity description was provided to the students as well as a live overview at the beginning of the semester. Over the course of nine weeks, student groups were required to present 18 summarized didactic lectures covering multiple therapeutics modules. A grading rubric was provided to the facilitators and one student group on a rotating schedule to evaluate each group. In addition, each presenting group was required to include at least two interaction questions at the end of the presentation and two random student groups were assigned each week to write one challenging question with references for each presentation. Student groups were required to email the presentations and questions to the facilitators to review and approve the presentation and question contents. Furthermore, a clinical faculty member from the KSU College of Pharmacy or clinical pharmacist from the university hospital, King Khalid University Hospital (KKUH), with expertise in the disease states presented was invited on a weekly basis as a guest facilitator. The guest facilitators would observe the presentations, ask questions, and provide beneficial comments and feedback. Facilitators and students were also allowed to ask additional questions and give recommendations.

#### 2.2.4 Capstone OSCE

A Capstone OSCE was designed to encompass an overview of all therapeutics modules covered throughout the curriculum. The Capstone OSCE was intended to serve as a final assessment for the CRQs and DSPs. As such, two settings were included: outpatient and inpatient, each with a standardized simulated patient. Ten exam rooms in the College of Medicine Simulation Center were reserved: five rooms each for the outpatient and inpatient interviews. Two clinical cases for each setting were developed and reviewed by the clinical faculty members. In addition, ten standardized patients were requested for the exam. The OSCE was shifted to an online format via Zoom due to the COVID-19 pandemic.

An OSCE overview session was provided to the students as well as a list of suggested guidelines and reading materials in the weeks prior to the exam. On the exam day, students were scheduled for 40-minute intervals with their assigned facilitators. The facilitators included clinical faculty members, teaching assistants, pharmacy residents, and Master of Clinical pharmacy interns. A brief overview was provided to all facilitators orally and the OSCE instructions as well as the cases were emailed to all facilitators prior to the OSCE. Students began with the patient chart review. The student reviewed the patient chart via blackboard, and the link was made available in blackboard for 5 min. The patient interview would follow and lasted for 10 minutes. The facilitator would play the role of the patient and provide subjective information based upon the scenario script in response to the students’ questions. The facilitator would simultaneously evaluate the student using the grading rubric for patient interview. The preparation stage followed the interview. The student was allowed 15 minutes to prepare a brief case presentation about the patient and formulate recommendations for all of the patient’s disease states. Students were allowed to use any references/resources available at their disposal. The final 10 minutes were dedicated to the physician consultation. The student would present a brief case/ Subjective, Objective, Assessment and Plan (SOAP) note orally with their recommendations. Facilitators would play the role of a physician based upon the key answers and use probing questions as needed to assess the students. The facilitator would simultaneously evaluate the student using the grading rubric for the physician consultation. The student was then given 50 minutes to write a SOAP note using any available resources based upon the patient interview and physician consultation and submit it via Blackboard.

#### 2.2.5 Basic biomedical sciences, pharmaceutical sciences, social/behavioral/administrative sciences review course

In addition to the review of the clinical topics, a one-day, 10-hour course was organized including expert KSU College of Pharmacy faculty and KKUH clinical pharmacy clinicians to deliver review sessions covering: Basic Biomedical Sciences, Pharmaceutics/ Biopharmaceutics/ Pharmacokinetics, Pharmacognosy and Dietary Supplements, Medicinal Chemistry, Sterile and Nonsterile Compounding, Social/Behavioral/Administrative Sciences, Pharmacology and Toxicology, Principles of biotechnology and its application, and Biostatistics. The course was scheduled to be held on the KSU campus and was free for all PPL-6 students as well as Bachelor of Pharmacy and Master of Clinical Pharmacy students. The review course was postponed at the beginning of the COVID-19 lockdown and was eventually canceled due to the continuation of the lockdown beyond the end of the semester.

#### 2.2.6 SPLE review survey

After completing the required SPLE preparation activities and their semester examinations, an anonymous, voluntary survey was used to assess the impact of these activities on student readiness levels and performance on the SPLE. A Google Forms® questionnaire was designed with 36 open- and closed-ended statements to assess student perceptions and opinions on the effectiveness of these activities on SPLE readiness levels. A 5-point Likert scale (ranging from “Strongly Disagree” to “Strongly Agree”) was used to quantify responses for the closed-ended questions. The questionnaire was distributed via email and WhatsApp® in March of 2021 when it was expected that the majority of students had taken the SPLE. Students were encouraged to complete the survey within one month. A reminder was sent via the class leaders after a month had passed. All data were expressed as means and standard deviations of each parameter.

### 2.3 Ethical considerations

This study was approved by the KSU College of Medicine Institutional Review Board (Project# E-21–5718).

### 2.4 Statistical analysis

All data were expressed analyzed using SPSS version26 and results were expressed as means and standard deviations of each parameter. P value < 0.05 indicated significant results.

## 3 Results

As shown in Table 1, a total of 127 Pharm.D. students were included in the study, of which 60% were females (n = 76). The average mock SPLE score was surprisingly low (55.8% ± 8.55) although students were in the 10th level- the last year of didactic courses prior to the start their advanced pharmacy practice experience (APPE) rotations. Nevertheless, their performance on the DSPs was better (91.3% ± 7.17) with a significantly higher average score for females compared to male students (95.2% ± 3.05 vs 86.6% ± 7.94, p < 0.001 respectively). Furthermore, the students performed exceptionally well on the Capstone OSCE (95.2% ± 6.90), with a significantly higher average score for the female students compared to their male counterparts (96.9% ± 4.09 vs 92.6% ± 9.11, p < 0.001, respectively). Additional details about students’ scores are provided in Table 1.

**Table 1:** Students‘ scores percentage.

**Final exam (Patient interview + Physician Consultation) (n)**	**Physician Consultation (n)**	**Patient interview (n)**	**Diseases State Presentations (n)**	**SPLE MOCK Pre-Exam (n)**	**Exam**
**95.2%** ± **6.90**	**91.5%** ± **9.56**	**96.2%** ± **6.91**	**91.3%** ± **7.17**	**55.8%** ± **8.55**	**Overall score (F + M)**
**(n = 127)**	**(n = 127)**	**(n = 127)**	(n = 114)**	(n = 122)*	
**92.6%** ± **9.11**	**87.1%** ± **11.66**	**94.1%** ± **9.25**	**86.6%** ± 7.94**	**55.2%** ± 8.57*	**Score (M)**
**(n = 51)**	**(n = 51)**	**(n = 51)**	**(n = 51)**	**(n = 50)**	
**96.9%** ± **4.09**	**94.5%** ± **6.40**	**97.7%** ± **4.27**	**95.2%** ± 3.05**	**56.3%** ± 8.56*	**Score (F)**
**(n = 76)**	**(n = 76)**	**(n = 76)**	**(n = 63)**	**(n = 72)**	
**Independent t-Test**					**Statistical test**
< **0.001**	< **0.001**	**0.004**	< **0.001**	**0.469**	**P value (F vs M)**

Data are expressed as Mean ± SD.

* 4 females and 1 male did not take the SPLE MOCK pre-exam.

** 13 female scores were excluded because their scores could not be determined.

The response rate for the SPLE questionnaire was approximately 50% with the majority of respondents being female (62.5%, n = 40) as shown in Table 2. The average GPA of responders was 4.7 ± 0.2 and 48.4% and 39.1% of them had “A” and “B” grades in the previous therapeutic courses taken throughout the college curriculum, respectively. Most students had taken and passed the SPLE on the first attempt (94.6%) with an average score of 635.7 ± 39.4 (∼79%). Of those, 78.6% scored above average on the clinical section, and approximately 83.9% indicated that they performed well on the clinical sciences domain as compared to the other areas **(**Table 3**)**. Over 60% of students recommended the SPLE CRQs activity because they believed that it helped in passing the SPLE **(**Table 4**).** Similarly, over 70% of participants indicated that the DSP activity was beneficial as well and hence recommended **(**Table 4**)**. Although 38% (n = 22) of students had participated in extra paid-courses preparation for SPLE, only 41% recommended them **(**Table 5**)**. These study aids ranged from 120 to 2000 Saudi Arabian Riyals (SAR) (32 – 533 USD) with an average of 325 SAR (87 USD). Further information about preparation courses is provided in Table 5.

**Table 2:** Survey participant demographics (n = 64).

**Question**	**Response**				
Gender	Male (n = 24; 37.5%)				
n = 64	Female (n = 40; 62.5%)				
Average age (years)	23.5 ± 0.6				
n = 64					
Average Student GPA (out of 5)	4.7 ± 0.2				
n = 63					
Average score in previous relevant therapeutic courses (%)?	**90**–**100**	**80**–**89.99**	**70.79.99**	**60**–**69.99**	< **60**
n = 64	48.40%	39.10%	9.40%	3.10%	0%

*The number within the parentheses is the corresponding percentage of responses.

**Table 3:** Student attempts and score of Saudi Pharmacist Licensure Examination (SPLE).

**Questions**	**Responses**			
Have you taken the Saudi Pharmacist Licensure Examination (SPLE) yet?	Yes	No		
n = 64	90.60%	9.40%		
How many times did you take the SPLE?	One time (89.7%)	Two times (10.3%)	Three times (0%)	Four times (0%)
n = 58				
Did you pass the SPLE?	Yes	No		
n = 58	96.60%	3.40%		
How did your scores compare in the clinical section with other test takers (based on the graph provided on your results)?	Above average	Average	Below average	
n = 56	78.60%	17.90%	3.60%	
Which area of the exam did you perform well (based on the graph provided on your results)?	Clinical science	Basic biomedical sciences	Pharmaceutical sciences (33.9%)	Social/behavioral/administrative sciences
n = 56	83.90%	41.10%		53.60%
Average score (on a scale of 200–800)	635.7 ± 39.4			
n = 56				
At which attempt did you obtain this score?	1st attempt	2nd attempt	3rd attempt	4th attempt
n = 56	94.60%	5.40%	0%	0%

*The number within the parentheses is the corresponding percentage of responses.

**Table 4:** SPLE preparation.

**Questions**	**Responses**			
Approximately how much time (in minutes) per week did it take you to complete the SPLE CRQs activity? n = 58	<30 min (34.5%)	30–60 min (39.7%)	60–120 min (13.8%)	More than 120 min (12.1%)
Would you recommend the SPLE CRQs activity for other test takers to pass the SPLE? n = 58	Yes (60.3%)	No (39.7%)		
Approximately how much time (in minutes) per week did it take you to complete the presentation activity? n = 57	<30 min (14%)	30–60 min (21.1%)	60–120 min (26.3%)	More than 120 min (38.6%)
Would you recommend the presentation activity for other test takers to pass the SPLE? n = 57	Yes (70.2%)	No (29.8%)		
Beside the SPLE CRQs and presentation activities, did you take another SPLE preparation course? n = 58	Yes (37.9%)	No (62.1%)		

*The number within the parentheses is the corresponding percentage of responses.

**Table 5:** SPLE Preparation course.

**Question**	**Response**	
What was the type of the course? n = 22	-Unspecified preparation course (45%)	
	-Dr. Jawzaa‘s preparation course (41%)	
	- Lessons and questions (4.6%)	
	- Summer course SASEM (4.6%)	
	- Lectures and educational summaries of the topics required in SPLE (4.6%)	
How much total time (in hours) did you spend on the course?	-The answers are not consistent and difficult to interpret	
	- some responses widely ranged from (0 to 168 hours) total studying hours	
	-Some students mentioned number of hours weekly (ranged from 3 to 20hrs/week) but did not specify the total studying weeks	
Would you recommend this course for other test takers to pass the SPLE? n = 22	Yes	No
	−40.90%	−59.10%
What other means did you use to prepare for the SPLE? [Hint e.g. online shared questions, YouTube channels, etc.] n = 22	-Youtube/telegram channels	
	-WhatsApp group	
	-Summaries for other pharmacists	
	-Books (such as NAPLEX-Rxprep)	
	- Self-learning	
	- Online training	
	- Questions	
Approximately how much money (in Saudi riyals) did you spend on materials/courses to prepare for the SPLE? n = 22	Ranged from 120 to 2000 SR with an average of 325 SR.	

Table 6 presents the Likert-scale based statements that were included in the questionnaire to get more insight about the overall impact of SPLE CRQs, DSPs, mock SPLE and Capstone OSCE on students’ preparation for the SPLE from their perspective. Although the SPLE CRQs did not appear to save their studying time, 56.3% of students agreed upon considering them more challenging than SPLE, which led to the use of other references to answer the questions occasionally. Additionally, 33.3% agreed that the DSP reduced the time spent on SPLE preparation, and 34.9% affirmed their benefit on preparing for the SPLE. With respect to mock SPLE, more students (60.9%) believed that it provided an idea of what to expect on the SPLE. Finally, despite the agreement of the majority of respondents on level of difficulty of patient case scenarios of the Capstone OSCE compared with the SPLE, they believed that it reduced their studying time and helped in performing well on the SPLE. Overall, the majority of students would recommend these activities be incorporated in the college of pharmacy curriculum in order to better prepare pharmacy graduates for the SPLE. To investigate factors associated with better SPLE performance among students, correlation analysis was performed and indicated that the average student score in therapeutic courses and student GPAs were associated with higher SPLE scores **(**Table 7**)**. The advantages and disadvantages regarding each activity from students’ perspectives are provided in Table 8.

**Table 6:** SPLE and OSCE (Likert scale).

**Statement**	**Strongly agree n (%)**	**Agree n (%)**	**Neutral n (%)**	**Disagree n (%)**	**Strongly Disagree n (%)**	**Not applicable for me n (%)**	**Mean**	**SD**
	**5**	**4**	**3**	**2**	**1**	**3**		
I always exerted my maximum personal effort when completing the SPLE CRQs (n = 64).	7 (10.9%)	23 (35.9%)	23 (35.9%)	8 (12.5%)	3 (4.7%)	–	3.36	1.00
I used/requested assistance when answering the questions from other classmates, textbooks, slides, guidelines, etc. (n = 64).	8 (12.5%)	28 (43.8%)	15 (23.4%)	6 (9.4%)	7 (10.9%)	–	3.38	1.16
I would retake the quizzes to improve my scores before submitting my results to the course instructors (n = 64).	7 (10.9%)	16 (25%)	21 (32.8%)	14 (21.9%)	6 (9.4%)	–	3.06	1.14
The SPLE CRQs questions were more difficult than the actual SPLE questions (n = 64).	25 (39.1%)	11 (17.2%)	17 (26.6%)	4 (6.3%)	1 (1.6%)	6 (9.4%)	3.86	1.03
I believe the weekly SPLE CRQs helped save me time studying for the actual SPLE (n = 64).	2 (3.1%)	7 (10.9%)	16 (25.0%)	18 (28.1%)	11 (17.2%)	10 (15.6%)	2.55	0.99
I believe that the SPLE CRQs helped me do well on the actual SPLE (n = 64).	3 (4.7%)	10 (15.6%)	20 (31.3%)	10 (15.6%)	12 (18.8%)	9 (14.1%)	2.72	1.09
I would recommend the SPLE CRQs activity to be incorporated in the College of Pharmacy curriculum (n = 64).	8 (12.5%)	24 (37.5%)	15 (23.4%)	11 (17.2%)	6 (9.4%)	–	3.27	1.17
I always exerted my maximum personal effort when completing the Disease State presentation activity (n = 63).	26 (41.3%)	21 (33.3%)	9 (14.3%)	4 (6.3%)	3 (4.8%)	–	4.00	1.12
I believe the Disease State presentation activity helped save me time studying for the actual SPLE (n = 63).	7 (11.1%)	14 (22.2%)	20 (31.7%)	8 (12.7%)	8 (12.7%)	6 (9.5%)	3.06	1.15
I believe that the Disease State presentation activity helped me do well on the actual SPLE (n = 63).	6 (9.5%)	16 (25.4%)	19 (30.2%)	6 (9.5%)	9 (14.3%)	7 (11.1%)	3.06	1.15
I would recommend the Disease State presentation activity to be incorporated in the College of Pharmacy curriculum (n = 63).	12 (19.0%)	30 (47.6%)	14 (22.2%)	5 (7.9%)	2 (3.2%)	–	3.71	0.97
The Mock SPLE exam was more difficult than the actual SPLE (n = 64).	6 (9.4%)	13 (20.3%)	18 (28.1%)	15 (23.4%)	5 (7.8%)	7 (10.9%)	3.00	1.07
The Mock SPLE exam gave me an idea of what to expect on the actual SPLE (n = 64).	13 (20.3%)	26 (40.6%)	13 (20.3%)	2 (3.1%)	3 (4.7%)	7 (10.9%)	3.69	0.96
I believe that the Mock SPLE exam helped me to identify the areas that I needed to focus on more when studying for the actual SPLE (n = 64).	8 (12.5%)	15 (23.4%)	23 (35.9%)	13 (20.3%)	5 (7.8%)	–	3.13	1.12
I believe that the Mock SPLE exam helped me do well on the actual SPLE (n = 64).	3 (4.7%)	13 (20.3%)	22 (34.4%)	8 (12.5%)	9 (14.1%)	9 (14.1%)	2.89	1.04
I would recommend the Mock SPLE exam to be incorporated in the College of Pharmacy curriculum (n = 64).	15 (23.4%)	31 (48.4%)	15 (23.4%)	3 (4.7%)	0 (0%)	–	3.91	0.81
The Capstone OSCE case scenarios were more difficult than the actual SPLE questions (n = 64).	3 (4.7%)	16 (25.0%)	19 (29.7%)	14 (21.9%)	5 (7.8%)	7 (10.9%)	2.97	0.99
I believe that preparing for the Capstone OSCE case scenarios helped save me time studying for the actual SPLE (n = 64).	2 (3.1%)	17 (26.6%)	15 (23.4%)	12 (18.8%)	10 (15.6%)	8 (12.5%)	2.83	1.09
I believe that the Capstone OSCE helped me do well on the actual SPLE (n = 64).	2 (3.1%)	19 (29.7%)	18 (28.1%)	9 (14.1%)	9 (14.1%)	7 (10.9%)	2.94	1.07
I would recommend the Capstone OSCE be incorporated in the College of Pharmacy curriculum (n = 64).	7 (10.9%)	28 (43.8%)	19 (29.7%)	9 (14.1%)	1 (1.6%)	–	3.48	0.93

*The number under the rating column indicates the number of students who picked that option and the number within the parentheses is the corresponding percentage of students.

**Table 7:** Correlation Analysis of student factors and SPLE score.

Correlation parameters		AGE	Average student score in relevant therapeutic courses	GPA	Gender (Being a male)	SPLE Score
AGE	Pearson Correlation	1	−0.150-	0.000	−0.046-	0.162
	Sig. (2-tailed)		0.238	0.997	0.717	0.232
	N	64	64	63	64	56
Average student score in relevant therapeutic courses	Pearson Correlation	−0.150-	1	0.555**	−0.371-**	0.343**
	Sig. (2-tailed)	0.238		0.000	0.003	0.010
	N	64	64	63	64	56
GPA	Pearson Correlation	0.000	0.555**	1	−0.507-**	0.518**
	Sig. (2-tailed)	0.997	0.000		0.000	0.000
	N	63	63	63	63	55
Gender (Being a male)	Pearson Correlation	−0.046-	−0.371-**	−0.507-**	1	−0.159-
	Sig. (2-tailed)	0.717	0.003	0.000		0.243
	N	64	64	63	64	56
SPLE Score	Pearson Correlation	0.162	0.343**	0.518**	−0.159-	1
	Sig. (2-tailed)	0.232	0.010	0.000	0.243	
	N	56	56	55	56	56

** Correlation is significant at the 0.01 level (2-tailed).

**Table 8:** Advantages and Disadvantages of teaching the course through SPLE CRQs and Mock SPLE*.

**Advantages**	**Disadvantages**	**Comments/suggestions**
Familiarity with all problems and how to solve them, deep thinking about the answer, and searching for answers through primary sources	Lack of pre-exam preparation and lack of knowledge of the test sections that need self-improvement	I suggest adding this course to the internship year, where the student is less busy and can focus on SPLE preparation
Focusing more on the student's weaknesses and giving a clear picture of the most important points that must be covered when studying for the SPLE test.	The questions focus only on the clinical aspect which represents only 35% of the test, while the test includes several other parts.	The topics should include all sections (clinical and nonclinical topics) of the test, not just the clinical section. From each section the student is given the most important information by cooperation with faculty in other departments
The presentations were very useful and the questions at the end of the presentations are an excellent idea to motivate the listeners to focus during the presentation.	The Presentation activity was a way of evaluating presentations rather than a way of learning	The weekly Quizzes should be close to the level of the SPLE test.
The mock exam is one of the most helpful experiences that helped me to understand the nature of the SPLE sections and the questions.	Taking the mock test once only. Not giving a detailed result of the mock test score, to know the strengths and weaknesses of the relevant parts.	Increasing the number of mock exams and distributing it over several sessions and allocating them to specific classes.
A comprehensive review and recall of all previously studied information particularly of the last year.	The clinical review (on the access pharmacy)/weekly Quizzes are more deep, difficult and not reflective for SPLE However, the SPLE focuses on the First Line treatment and contraindications.	Instead of an assignment, there will be an activity every week or two in the lab with a lecturer specialized in the topic being discussed where questions and answers are discussed with the students.
Training and Psychological preparation for SPLE		
The OSCE Online Test was the best feature of it! Its arrangement, organization, and feeling that it is a real test is enjoyable		

* Only most important and relevant comments were presented.

## 4 Discussion

To the best of our knowledge, this is the first study that discusses a comprehensive preparatory module for the SPLE and the impact on students’ perceptions, readiness, and performance on the SPLE exam.

The average percentage score of the SPLE mock pre-exam was 55.8%, while the passing score on the SPLE is approximately 67%. Interestingly, students who took both exams reported higher scores in the SPLE compared to SPLE mock pre-exam [79.4% (635.7 ± 39) vs 55.8%, respectively]. Additionally, despite being at the same level of difficulty, a higher proportion of students reported that the SPLE mock pre-exam was more difficult than the SPLE exam. Although the questionnaire respondents had high GPAs and high average scores in therapeutics courses, which suggests a sampling bias and might have influenced their beliefs about the difficulty level of both exams, the impact of DSPs and the Capstone OSCE on the SPLE results cannot be neglected. The majority agreed and attributed their exceptional performance on the SPLE to these activities. This resonates with the low level of difficulty of the SPLE reported by the students as a consequence of their well-preparation. These results are partially supported by the study published by Karimi and colleagues in 38 third year Pharm.D. students (P3) which showed no correlation between their prepharmacy GPA and their performance on the NAPLEX. (Karimi et al., 2014).

In our study, 96.6% of the students who responded to the survey passed the SPLE and 89.7% of students who passed it scored 635.7 ± 39.4 (∼79.4%) on their first attempt. According to the public data available on the SCFHS website, we found that the passing rate of applicants in 2019 SPLE was 100% (111/111). (Saudi Commission for Health Specialties, 2022) In 2020, the passing rate was 99% (112/113). Despite this drop, the average and maximum passing scores of the students in 2020 were 599 and 710, respectively, which were higher than 2019 which were 595 and 698, respectively. These rates and scores are based on all attempts of students. To avoid the impact of several attempts taken by students on these scores, we limited our analysis to the first attempt only. Interestingly, we found that the passing rate in 2019 was 100% (95/95) with average and max pass score of 601 and 698, respectively. Whereas in 2020, it was 99% (91/92) with average and max pass score of 604 and 710, respectively. Collectively, this indicates that incorporation of these activities could potentially enhance student’s performance and improve the curriculum learning outcomes, particularly passing the SPLE with high scores. Furthermore, the high passing rate on the first attempt may be attributed to the location of the college within an academic health center, namely KKUH. This has been shown previously by William and colleagues that students from pharmacy schools located within academic health centers had higher NAPLEX first-time pass rates compared to students from schools not located in academic health centers. (Williams et al., 2019) Intriguingly, not only did the students perform well on the SPLE exam, but 78.6% reported higher average scores in the clinical science domain compared to the overall average score of test takers on this section according to the SPLE statement of results released to each candidate by the SCFHS. This could be related to the type of SPLE activities adopted in this course, namely SPLE CRQs and the Capstone OSCE, that primarily focused on improving the clinical knowledge of students. Similar results have been reported by Elder and colleagues where patient case work-up/presentation and associated critical thinking activities significantly predicted 31.1% of variability in the NAPLEX scores. (Elder et al., 2019).

Approximately 50% and 66.6% of the SPLE exam takers suggested integrating the SPLE CRQs and DSPs in the college curriculum, respectively. This was reiterated by students who passed the SPLE as 60% and 70% of them recommended these activities, respectively, for prospective SPLE candidates due to the benefit noted on the SPLE performance. To further support this, Karimi and colleagues demonstrated that students participated in a longitudinal pharmacotherapeutic poster presentation activity performed significantly higher on the NAPLEX and 83% highlighted the benefit of that on their preparation for the NAPLEX. (Karimi et al., 2014) Additionally, 71.8% and 55% of the SPLE exam takers in our study suggested that the SPLE mock pre-exam and Capstone OSCE be incorporated in the college curriculum, respectively, as they positively impacted their SPLE scores. Interestingly, this is supported by Stewart et al. who demonstrated that integrating and conducting a mock dental board exam and clinical procedures for dental students was significantly correlated with the performance and passage of the Florida dental licensure exam. (Stewart et al., 2004) Moreover, and despite the absence of the overall impact on the NAPLEX, Lahoz et al. showed a significant improvement in NAPLEX review exam scores. Pharm.D students were mandated to take the NAPLEX review exams three times in a 2-year period as part of a longitudinal comprehensive NAPLEX review program. (Lahoz et al., 2010) Overall, although our students have widely varied in their opinions regarding adding various SPLE-related activities into the college curriculum, a higher proportion preferred incorporating the SPLE mock pre-exam (n = 46, mean = 3.91 ± 0.81) and DSPs (n = 42, mean = 3.71 ± 0.97) compared to the Capstone OSCE (n = 35, mean = 3.48 ± 0.93) and SPLE CRQs (n = 32, mean = 3.27 ± 1.17). The recommendation to incorporate a mock SPLE exam in the curriculum was in spite of the fact that student opinions were relatively similar in their perspectives regarding whether it helped them to do well on the actual SPLE. It may be speculated that the reason is that the students support the idea of using the mock SPLE exam; however, the content of the mock SPLE may need to be modified in order to prepare students for the actual exam and/or a post-SPLE mock exam is needed to better assess student readiness. In regards to the external courses and programs, only a few students (n = 22 of 58) reported using them and the majority (59%) did not recommend enrolling in or utilizing them. Nevertheless, the financial burden on students from these programs may have influenced their opinions.

With respect to student comments/suggestions obtained from the questionnaire, some students stated that the SPLE CRQs, designed specifically for the NAPLEX exam, were more advanced, intended to test a higher level of critical thinking and were not reflective of the questions on the SPLE. Although this might be true to some extent, preparation with mentally stimulating questions is considered advantageous because they may improve one's confidence and competency and make them ready for the real-world practice, which requires critical thinking. Other students complained of the burden of the review quizzes during the course as well as the requirement to achieve a minimum score to pass them which distracted them from focusing on the benefits of the activity. Despite that, one of the suggestions was to shift these activities to the APPE internship year so students could benefit more during their preparation for the SPLE. Although this could potentially be adopted during the APPE rotations, a variation in the level of difficulty of the activities that students would be exposed to in their internships and the time dedicated to complete these activities might arise as a result of the differences in the preceptors’ expectations and the type of rotation. However, all these potential obstacles were overcome by integrating these activities within this course.

Correlative analyses were conducted to investigate student factors that influence average therapeutic scores, GPA and SPLE scores. Our analyses showed a significantly higher GPA with female gender. In addition, higher average scores in therapeutic courses were significantly associated with higher GPA as well as the female gender. Further, there were no significant associations between SPLE score and age or gender, which contradicts the findings of Alhifany et al. that suggested that being male was associated with significantly higher SPLE scores (Alhifany et al., 2020). More importantly, high average scores in therapeutic courses and GPA among our students were significantly associated with higher SPLE scores, which aligns with Alhifany’s findings that found a significant association between SPLE scores and pharmacology/therapeutics grades, pharmacy GPA and preparatory year GPA. Furthermore, our observations are in agreement with Chisholm-Burns et al. and Allen and Diaz who reported a significantly higher total NAPLEX score being associated with elevated GPA (Chisholm-Burns et al., 2017, Allen and Diaz, 2013).

There were several limitations of this study. First, many modifications were made to the SPLE review activities due to the COVID-19 pandemic. The live SPLE review session to cover the other domains, namely Basic Biomedical Sciences, Pharmaceutical Sciences, Social/Behavioral/Administrative Sciences, was canceled; the shortened semester resulted in less time to complete the activities; and the OSCE and some of the DSPs were changed to a virtual format. Therefore, the students were not exposed to all of the SPLE domains.

Second, the clinical review questions were based upon the NAPLEX review questions/quizzes which is a similar, yet different exam; therefore, the level of difficulty and relevance may have differed. In addition, students were given random CRQs which differed from their colleagues. Beside that, and as confirmed by the questionnaire, students took the quizzes multiple times to achieve a passing score. Thus, their baseline knowledge could not be assessed, and their scores were not be used in the results since they do not accurately reflect their real performance.

Third, the results of the study cannot be generalized to other Saudi universities since these activities were conducted at one Saudi university only and the questionnaire respondents represented approximately half of the students included in the study.

To overcome these limitations, future studies should incorporate similar activities as planned prior to the advent of the COVID-19 pandemic and should be conducted at multiple Saudi universities. Furthermore, the CRQs should be more similar to the SPLE exam. In addition, all students’ demographics should be accessed and analyzed including SPLE scores, GPA, age, etc.

## 5 Conclusion

SPLE review courses are expected to become in higher demand in Saudi Arabia. Prospective graduates from Saudi universities may benefit from college of pharmacy-organized SPLE reviews. Based on this study’s findings, a comprehensive review course including a mock SPLE and disease state presentations is recommended. In addition, SPLE review lectures, CRQs, and a Capstone OSCE may provide additional benefit.

## Declaration of Competing Interest

The authors declare that they have no known competing financial interests or personal relationships that could have appeared to influence the work reported in this paper.

## Figures and Tables

**Table:** 

**Week #**	**Student Presentation Topics/Review Modules**
**1**	Introduction to the course and formation of groups
**2**	Capstone OSCE / SPLE/ Presentation format
**3**	Cardiovascular Module Review
**4**	Student-presented topic 1 (Hypertension, Dyslipidemia) Respiratory Module Review
**5**	Student-presented topic 2 (Asthma, COPD) Renal and Nutritional Disorders Module Review
**6**	Student-presented topic 3 (Acute Kidney Injury, Chronic Kidney Disease) Gastrointestinal Disorders Module Review
**7**	Student-presented topic 4 (GERD, Hepatitis C) Bone and Joint Disorders Module Review
**8**	Student-presented topic 5 (Rheumatoid arthritis, Osteoporosis) Endocrinologic Disorders Module Review
**9**	Student-presented topic 6 (Diabetes, Hypothyroidism) Psychiatric Module Review
**10**	Student-presented topic 7 (Schizophrenia, Depression) Neurologic Module Review
**11**	Student-presented topic 8 (Epilepsy, Alzheimer’s) Infectious Diseases Module Review
**12**	Student-presented topic 9 (Lower Respiratory Tract Infections, HIV) Immunologic/Hematologic/ Oncologic Module Review
**13**	**FINAL CAPSTONE OSCE**
